# Optimizing conditions for *E*-and *Z*-ajoene formation from garlic juice using response surface methodology

**DOI:** 10.1002/fsn3.148

**Published:** 2014-08-04

**Authors:** Miyoung Yoo, Sanghee Lee, Sunyoung Kim, Dongbin Shin

**Affiliations:** Korea Food Research InstituteSeongnam-si, 463-746, Korea

**Keywords:** Ajoene, High-performance liquid chromatography, oil-macerated garlic, response surface methodology

## Abstract

The optimum conditions for the formation of *E*- and *Z*-ajoene from garlic juice mixed with soybean oil were determined using response surface methodology. A central composite design was used to investigate the effects of three independent variables temperature (°C, *X*_1_), reaction time (hours, *X*_2_), and oil volume (multiplied by weight, *X*_3_). The dependent variables were *Z*-ajoene (*Y*_1_) and *E*-ajoene (*Y*_2_) in oil-macerated garlic. The optimal conditions for *E*- and *Z*-ajoene using ridge analysis were 98.80°C, 6.87 h, and weight multiplied by weight 2.57, and 42.24°C, 9.71 h, and weight multiplied by weight 3.08, respectively. These conditions resulted in *E*- and *Z*-ajoene compound predicted values of 234.17 and 752.62 *μ*g/g from garlic juice, respectively. The experimental values of *E*- and *Z*-ajoene were 222.75 and 833.59 *μ*g/g, respectively. The estimated maximum values at the predicted optimum conditions were in good agreement with experimental values.

## Introduction

Garlic, a member of the Liliaceae family, is a common foodstuff and medicinal food that is used to improve human health in many areas of the world (Hacıseferoğulları et al. 2005). Pharmacological studies have previously reported that garlic has many health benefits including anticancer, antithrombotic, antiviral, and antioxidant activities (MacDonald and Langler 2000; Wua et al. 2001; Ariga and Seki 2006; Hunter et al. 2008). The major causal compounds providing these beneficial effects are *S*-allyl-l-cysteine, thiosulfinates (allicin), ajoene, and other volatile sulfur-containing compounds (diallyl sulfide, diallyl disulfide, diallyl trisulfide, etc.). Thus, garlic cloves are commonly used as a medication by a large portion of the world, especially in Eastern Europe and Asia, while garlic pill supplements are popular in western Europe and are growing in popularity in the United States (Lawson and Bauer 1998).

Ajoene [(*E*, *Z*)-4,5,9-trithiadodeca-1,6,11-triene 9-oxide] was identified as an oxygen-containing transformation product of allicin in oil-macerated garlic extract by Block (1985). Ajoene, which consists of two isomers, *trans E*- and *cis Z*-forms, is not found in garlic bulbs, but is mainly formed by the incubation of garlic pulp in polar solvents (acetone, water, ethanol). Ajoene is also found in commercial oil-macerated garlic products. *Z*-ajoene is the main isomer formed when ajoene is prepared in nonpolar solvents such as vegetable oil, hexane, and ether whereas *E*-ajoene is the dominant isomer formed when ajoene is prepared in polar solvents such as alcohol and acetone. Commercial products typically have an *E*/*Z* ratio of ∼2, with a range of 0.6–2.5 (Iberl et al. 1990a; Lawson et al. 1991). The *E*/*Z* ratio and yield depend on the polarity of the solvent system, reaction condition, and the type of fatty acids that are present during processing. It is known that the *Z*-isomer has a strong bioactivity compared to *E*-ajoene, while the *E*-isomer is more stable than the *Z*-isomer during storage.

The best known biological activities of ajoene include its anti-microbial and cholesterol-lowering actions. Additionally, ajoene compounds appears to have potent inhibitory effects on platelet activation, platelet binding to damaged blood vessel walls and thrombus formation (Apitz-Castro et al. 1986; Lawson et al. 1992; Hassan 2004). Ajoene has also been reported to reduce the risk of developing skin and hepatic tumors that are associated with cancer (Hattori et al. 2001; Nishikawa et al. 2002).

Therefore, ajoene compounds are expected to have health benefits when used as nutraceutical food materials. However, most of the studies of the ajoene compounds have been focused on the identification, quantification, and biological properties of ajoene. When ajoene compounds are used in functional materials, the conditions for the maximum formation of ajoene from oil-macerated garlic are of great importance for evaluating the biological quality of garlic products. Many factors such as temperature, reaction time value, and the amount of edible oil influenced ajoene formation in garlic extract (Hibi 1997). Because no studies have been conducted concerning ajoene formation and conditions using response surface methodology (RSM), it is worth studying the optimum conditions of ajoene formation from oil-macerated garlic to determine the quality of oil-macerated garlic products containing ajoene.

RSM is a useful technique for analyzing interactions among factors and, exploring the relationships between the response and the independent variables. It is a collection of statistical and mathematical techniques that has been used for developing, improving, and optimizing various processes (Mayers and Montgomery 2002; Baş and Boyacı 2007). It provides relevant information over a short period of time without the need to perform a large number of experiments (Baş and Boyacı 2007). As a powerful statistical tool, RSM has been successfully used in various fields of food chemistry for studies such as the optimization of anthocyanin hydrolysis from red wine and the optimization of the solvent extraction of phenolic compounds from beans and other plants (Liyana-Pathirana and Shahidi 2005; Wang et al. 2007; Pinho et al. 2011).

The objective of the present study was to investigate the effects of temperature (*X*_1_, °C), reaction time (*X*_2_, hours) and oil volume (*X*_3_, multiplied by weight) on the formation of ajoene from oil-macerated garlic, and to optimize these main parameters by considering two responses using RSM.

## Experimental

### Materials

Ajoene compounds were purchased from Medigen (Daejeon, Korea) and 2-propanol, *n*-hexane and ethyl acetate were obtained from J.T. Baker (Paris, KY). Deionized water (DW) was purified through a Milli-Q system (Millipore, Bedford, MA) for all sample preparations and mobile phases. All other chemicals and solvents used were of high-performance liquid chromatography (HPLC) grade.

### Preparation samples

Garlic bulbs harvested in 2010 were purchased from a cultivator of Shinan in Korea. Soybean oil was obtained from a local market (Seongnam, Korea). Fresh garlic juice was prepared using a KAISO DH850 laboratory blender (OSCAR, Kimhae city, Korea) was used to determine the ajoene levels. Soybean oil was used to macerate the garlic juice and the samples were mixed using a vortex mixer (Thermo Scientific, Dubuque, IA) for 1 min and by ultra sonication (Baronsonic, Danbury, CT) for 20 min. The mixture was then incubated at various conditions for the complete formation of ajoene (Table 1). The mixed samples were centrifuged (Brinkman Instruments, Westbury, NY) at 3220 *g* for 5 min. Five milliliters of the supernatant was extracted with ethyl acetate and analyzed by HPLC. Ajoene analysis was modified according to Yoo et al. (2012).

**Table 1:** Uncoded and coded independent variables used in RSM.

		Code variable levels				
Symbol	Independent variables	−2	−1	0	1	2
*X*_1_	Temperature (°C)	20	40	60	80	100
*X*_2_	Time (hours)	2	4	6	8	10
*X*_3_	Oil volume (multiplied by weight)	1	2	3	4	5

### Experimental design for response surface methodology

RSM was applied to optimize the conditions for ajoene formation from fresh garlic juice reacted with soybean oil. The experimental design was developed using central composite design (CCD) (Khuri and Mukhopadhyay 2011). The CCD in the experimental design consisted of 23 factorial points, 6 axial points (*α* = 2), and 3 replicates of the central point. Table 1 shows the range and center point values of three independent variables. The three independent variables were temperature (*X*_1_, °C), reaction time (*X*_2_, hours), and oil volume (*X*_3_, multiplied by weight) and the dependent variables were *E*-ajoene (*Y*_1_, *μ*g/g) and *Z*-ajoene (*Y*_2_, *μ*g/g) in oil-macerated garlic. The content of ajoene in the oil-macerated garlic was selected as the dependent variable for the combination of the independent variables as shown in Table 2. The variables were coded according to the following equation:


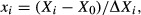


where *x_i_* is the coded value of an independent variable, *X*_*i*_ is the real value of an independent variable, *X*_0_ is the real value of an independent variable at the center point, and Δ*X*_*i*_ is the step change value. The RSM procedure and experimental data were analyzed using Statistical Analysis System software (Version 8.02; SAS Institute Inc., Cary, NC). The quadratic polynomial equation is:


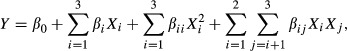


where *Y* is the dependent variable of ajoene content; *β*_0_ is a constant, *β*_*i*_, *β*_*ii*_ and *β*_*ij*_ are regression coefficients and *X*_*i*_, *X*_*j*_ are levels of the independent variable. The equations represent the linear, quadratic and interaction effects of the variables. The statistical analysis of the model was performed by analysis of variance (ANOVA). The significance of each term in the polynomial was assessed statistically by the *F*-value at a probability (*P*) of 0.001, 0.01 or 0.05. The three-dimensional response plots were generated using Maple software (Maple 7.0; Waterloo Maple Inc., Waterloo city, ON, Canada).

**Table 2:** Response surface design and experimental data.

	Code level of variables^1^			Response^2^	
Run no.	*X*_1_	*X*_2_	*X*_3_	*Y*_1_	*Y*_2_
1	40 (−1)	4 (−1)	2 (−1)	537.70	25.89
2	40 (−1)	4 (−1)	4 (1)	488.75	23.08
3	40 (−1)	8 (1)	2 (−1)	633.34	32.70
4	40 (−1)	8 (1)	4 (1)	510.65	28.82
5	80 (1)	4 (−1)	2 (−1)	515.23	222.74
6	80 (1)	4 (−1)	4 (1)	418.17	209.76
7	80 (1)	8 (1)	2 (−1)	284.98	256.42
8	80 (1)	8 (1)	4 (1)	243.54	213.08
9	60 (0)	6 (0)	3 (0)	568.27	82.99
10	60 (0)	6 (0)	3 (0)	599.57	85.42
11	60 (0)	6 (0)	3 (0)	584.30	84.99
12	100 (2)	6 (0)	3 (0)	35.48	123.78
13	20 (−2)	6 (0)	3 (0)	323.72	15.13
14	60 (0)	10 (2)	3 (0)	720.79	87.05
15	60 (0)	2 (−2)	3 (0)	644.90	37.17
16	60 (0)	6 (0)	5 (2)	507.80	46.08
17	60 (0)	6 (0)	1 (−2)	162.62	58.29

1 *X*_1_ is temperature (°C); *X*_2_ is time (hours); *X*_3_ is oil volume (multiplied by weight).

2 *Y*_1_ is *Z*-ajoene content; *Y*_2_ is *E*-ajoene content (*μ*g/g of garlic juice).

### Ajoene compound analysis

Ajoene analysis was determined according to the procedure described by Yoo et al. (2012) using the HPLC system.

## Results and Discussion

### Fitting model

The effect of three variables, temperature (*X*_1_: 20–100°C), reaction time (*X*_2_: 2–10 h) and oil volume (*X*_3_: weight multiplied by 1–5), were investigated in this study. These factors were selected during a preliminary study for the highest formation of *E*- and *Z*-ajoene. The two responses of interest were formation of *Z*-ajoene (*Y*_1_) and *E*-ajoene (*Y*_2_). The results of 17 runs using CCD are shown in Table 2, which included the response surface design and the observed responses. In addition, the range of *Z*-ajoene and *E*-ajoene yields was 35.48–720.79 and 15.13–256.42 *μ*g/g of garlic juice, respectively. The significance of each coefficient was determined using the *F*-test and *P*-value. Lack-of-fit was also included to check the quality of the fitted models.

Table 3 shows the result of fitting two quadratic models to the yield of ajoene from oil-macerated garlic. All the independents variables, temperature (*X*_1_, °C), reaction time (*X*_2_, hours) and oil volume (*X*_3_, multiplied by weight) have positive linear responses in two surface models (*Y*_1_ and *Y*_2_). The coefficients of determination (*R*^2^) for *Y*_1_ and *Y*_2_ were 0.8697 and 0.6508, respectively. These results indicate that the model for *Y*_1_ is suitable to represent the real relationships among the selected parameters but the model for *Y*_2_ is unsuitable. The values of *R*^2^ for *Y*_1_ and *Y*_2_ were significant at *P* = 0.05 and 0.5, respectively. We focused our experimental design on *Z*-ajoene formation in laboratory produced oil-macerated garlic because *Z*-ajoene exhibited higher biological activity such as antimicrobial activity than *E*-ajoene (Yoshida et al. 1998).

**Table 3:** Polynomial equation calculated by response surface methodology.

Response	Polynomial equation	*R*^2^	*P*-value
*Y*_1_	*Y* = −1026.189 + 35.379*X*_1_ + 21.892*X*_2_ + 389.138 *X*_3_ − 0.252*X*_1_² − 1.633*X*_1_*X*_2_ + 6.269*X*_2_² + 0.207*X*_1_*X*_3_ − 1.133*X*_2_*X*_3_ − 61.834*X*_3_²	0.86	0.0258
*Y*_2_	*Y* = 246.147 + 3.974*X*_1_ + 14.988*X*_2_ − 57.837*X*_3_ − 0.003*X*_1_² + 0.076*X*_1_*X*_2_ − 0.751*X*_2_² − 0.310*X*_1_*X*_3_ − 1.965*X*_2_*X*_3_ − 5.484*X*_3_²	0.65	0.3194

*Y*_1_ is *Z-*ajoene content; *Y*_2_ is *E*-ajoene content (*μ*g/g of garlic juice).

### Analysis of variance

The statistical significance of response surface models was evaluated by ANOVA. Tables 4 and 5 show the ANOVA results for the models that explain the response of two dependent variables, *Y*_1_ (*Z*-ajoene content) and *Y*_2_ (*E*-ajoene content). The quadratic term for *Y*_1_ was highly significant at the 99% probability level, whereas the linear terms of all dependent variables and the cross-product terms of *Y*_1_ and *Y*_2_ were not significant at the 95% probability level. The total regression model for *Y*_1_ was significant at the 95% probability level and the model for *Y*_2_ was not significant at the 95% probability level. The results of the lack-of-fit test, which indicates the fitness of the model, demonstrated that the dependent variable *Y*_1_ was significant at the 95% probability level. However, the lack-of-fit test for *Y*_2_ did not result in a significant (*P* < 0.05).

**Table 4:** ANOVA parameter for dependent variables.

Responses^1^	Regression	df	SS	*R*^2^	*F*-value	*P*-value
*Y*_1_	Linear	3	113,385	0.20	3.37	0.0843
	Quadratic	3	333,760	0.60	9.91	0.0065
	Cross product	3	34,294	0.06	1.02	0.4402
	Total model	9	481,439	0.86	4.76	0.0258
*Y*_2_	Linear	3	65,475	0.64	4.27	0.0519
	Quadratic	3	636.80	0.01	0.04	0.9877
	Cross product	3	505.99	0.00	0.03	0.9912
	Total model	9	66,618	0.65	1.45	0.3194

df, degrees of freedom; ss, sum of squares.

1 *Y*_1_ is Z-ajoene content; *Y*_2_ is E-ajoene content (*μ*g/g of garlic juice).

**Table 5:** ANOVA for the response surface model for ajoene formation.

Responses^1^	Source	df	SS	MS	*F*-value	*P*-value
*Y*_1_	Lack of fit	5	78,106	15,621	63.77	0.0155
	Pure error	2	489.94	244.97		
	Total error	7	78,596	11,228.00		
*Y*_2_	Lack of fit	3	35,745	7148.91	4241.31	0.0002
	Pure error	3	3.37	1.69		
	Total error	3	35,748.00	5106.84		

df, degrees of freedom; ss, sum of squares; MS, mean square.

1 *Y*_1_ is *Z*-ajoene content; *Y*_2_ is *E*-ajoene content (*μ*g/g of garlic juice).

### Optimization

The 3D plots of ajoene processed from oil-macerated garlic as a function of temperature (*X*_1_, °C), reaction time (*X*_2_, hours), and oil volume (*X*_3_, multiplied by weight) showed the optimum conditions. The optimal conditions included coded and uncoded values of each dependent variable (*Y*_1_ and *Y*_2_), and these conditions are shown in Table 6. According to the canonical analysis of the response surface, *Y*_1_ and *Y*_2_ had negative and positive values. Therefore, the stationary points were saddle points. A ridge analysis was performed to determine the critical levels of the design variables that produce the maximum response. The optimized values of the temperature (*X*_1_), reaction time (*X*_2_), and oil volume (*X*_3_) had the coded ranges of −0.1590 to −5.4673, −0.1184 to −5.0474, and 0.0929–4.6509, respectively.

**Table 6:** ANOVA of the factor obtained from ridge analysis of the response surface for ajoene formation.

	Analysis of variance					Critical values	
Response	df	SS	MS	*F*-value	*P-*value	Coded^1^	Uncoded
*Z-ajoene* (*Y*_1_)							
Temperature (*X*_1_, °C)	4	334,003	83,501	7.44	0.0116	−0.1590	53.6390
Time (*X*_2_, hours)	4	47,482	11,871	1.06	0.4434	−0.1184	5.5263
Oil volume (*X*_3_, multiplied by weight)	4	83,269	20,817	1.85	0.2234	0.09292	3.1858
*E-ajoene* (*Y*_2_)							
Temperature (*X*_1_, °C)	4	64,013	16003	3.13	0.0894	−5.4673	−158.6978
Time (*X*_2_, hours)	4	1766.17	441.54	0.09	0.9839	−5.0474	−14.1897
Oil volume (*X*_3_, multiplied by weight)	4	1491.46	372.87	0.07	0.9882	4.6509	12.3019

df, degrees of freedom; ss, sum of squares; MS, mean square.

1 Critical values obtained from ridge analysis.

### Interpretation of response surface models

**Figure 1:**
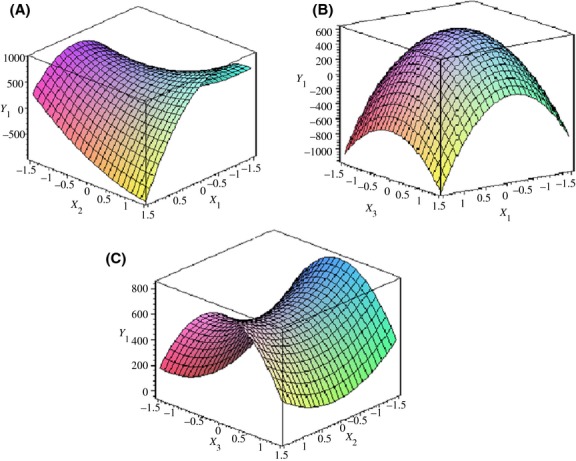
Response surface plot for optimization of *Z*-ajoene formation from oil-macerated garlic. *X*_1_ (time, hours), *X*_2_ (temperature, °C) and *X*_3_ (oil volume, multiplied by weight). *Y*_1_ (*Z*-ajoene content of *μ*g/g of garlic juice). The interaction between (A) temperature and time, (B) temperature and oil volume, (C) time and oil volume.

**Figure 2:**
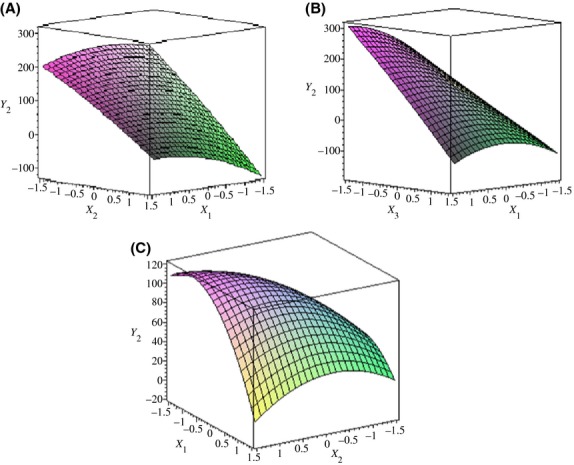
Response surface plot for optimization of *E*-ajoene formation from oil-macerated garlic. *X*_1_ (time, hours), *X*_2_ (temperature, °C), and *X*_3_ (oil volume, multiplied by weight). *Y*_2_ (*E*-ajoene content of *μ*g/g of garlic juice). The interaction between (A) temperature and time, (B) temperature and oil volume, (C) time and oil volume.

The 3D response surface models of the contents of *E*- and *Z*-ajoene are given in Figures 1, 2. The relationship between independent variables and dependent variables was drawn as a three dimension plot using the Maple program. Figure 1 shows the effect of independent variables on *Y*_1_ (*Z*-ajoene formation). When the coded values of two independent variables (*X*_1_ and *X*_3_) variables were close to zero, the *Z*-ajoene content increased. The temperature (*X*_1_) and oil volume (*X*_3_) parameters were statistically significant with temperature (*X*_1_) having the greatest effect. The *Z*-ajoene content decreased with an increase in reaction temperature 60–90°C, because it was gradually isomerized to *E*-ajoene at a higher temperature (Naznin et al. 2008). Table 6 explains the variance of temperature (*X*_1_), time (*X*_2_), and oil volume (*X*_3_) independent variables. Time had no significant effect on ajoene formation in oil-macerated garlic. Iberl et al. (1990a,b) found that the storage time of the reaction had little influence on ajoene formation. Figure 2 depicts the influence of temperature, time, and oil volume on *Y*^2^ (*E*-ajoene formation). It can be seen that temperature (*X*_1_) has a linear effect. Increases of temperature resulted in higher *E*-ajoene content in oil-macerated garlic. Additionally, time did not seem to affect *E*-ajoene formation in the selected range, whereas oil volume exerted a linear effect as shown in Figure 2B. Therefore, the reaction temperature setting could be the key factor in the formation of ajoene.

### Verification of predicted values

Based on the above findings, an optimization study was performed to evaluate the optimal operating conditions for individual responses. Table 7 shows the optimal conditions for each response with the predicted and experimental values. The optimal conditions for *Z*-ajoene formation were temperature of 45.25°C, reaction time of 9.71 h, and oil volume equivalent to weight multiplied by 3.08, whereas the optimal conditions for *E*-ajoene were formation temperature of 98.08°C, reaction time of 6.87 h, and oil volume equivalent to the weight multiplied by 2.57. The actual values that were repeated three times were *Z*-ajoene formation = 833.59 (*μ*g/g of garlic juice) and *E*-ajoene formation = 225.75 (*μ*g/g of garlic juice) as compared to the predicted values: *Z*-ajoene formation = 752.62 (*μ*g/g of garlic juice) and *E*-ajoene formation = 234.17 (*μ*g/g of garlic juice). The actual and predicted values were similar. Therefore, the estimated response surface model can be used to optimize the process of *Z*-ajoene formation from oil- macerated garlic.

**Table 7:** Predicted and experimental values under optimum conditions based on combination of responses.

	Independent variables^2^					
Responses^1^	*X*_1_	*X*_2_	*X*_3_	Stationary point	Predicted value^3^	Experimental value^4^
*Y*_1_	45.25	9.71	3.08	Saddle	752.62	833.59 ± 59.1
*Y*_2_	98.08	6.87	2.57	Saddle	234.17	225.75 ± 9.7

1 *Y*_1_ is *Z*-ajoene content; *Y*_2_ is *E*-ajoene content (*μ*g/g of garlic juice).

2 *X*_1_ is temperature (°C); *X*_2_ is Time (hours); *X*_3_ is oil volume (multiplied by weight).

3 Predicted using ridge analysis of response surface quadratic model.

4 Mean ± standard deviation of triplicate determination.

## Conclusions

RSM is a useful tool for establishing the optimum conditions for the formation of ajoene compounds from oil-macerated garlic. Using CCD, three dependent variables temperature, time and oil volume affect the ajoene yield. Temperature was determined to have the most significant effect on ajoene formation in garlic juice. Additionally, temperature, time, and oil volume were fit to quadratic models of ajoene formation. The maximum *Z*-ajoene content was 752.62 *μ*g/g of garlic juice at 45.25°C, 9.71 h and an oil volume equivalent to the weight multiplied by 3.08. The optimized conditions for *E*-ajoene was 98.08°C, 6.87 h and an oil volume equivalent to the weight multiplied by 2.57. Under these conditions, the content of *E*-ajoene was 234.17 *μ*g/g of garlic juice.
